# Rapid preoperative predicting tools for 1-year mortality and walking ability of Asian elderly femoral neck fracture patients who planned for hip arthroplasty

**DOI:** 10.1186/s13018-021-02605-0

**Published:** 2021-07-16

**Authors:** Guangtao Fu, Mengyuan Li, Yunlian Xue, Hao Wang, Ruiying Zhang, Yuanchen Ma, Qiujian Zheng

**Affiliations:** 1 Department of Orthopedics, Guangdong Provincial People’s Hospital, Guangdong Academy of Medical Sciences, Guangzhou, Guangdong Province People’s Republic of China; 2 Division of Statistics, Guangdong Provincial People’s Hospital, Guangdong Academy of Medical Sciences, Guangzhou, Guangdong Province People’s Republic of China

**Keywords:** Femoral neck fracture, Nomograms, Mortality, Walking ability, Prognosis prediction

## Abstract

**Background:**

Femoral neck fractures in elderly patients typically warrant operative treatment and are related to high risks of mortality and morbidity. As early hip arthroplasties for elderly femoral neck fractures are widely accepted, rapid predicting models that allowed quantitative and individualized prognosis assessments are strongly needed as references for orthopedic surgeons during preoperative conversations.

**Methods:**

Data of patients aged ≥ 65 years old who underwent primary unilateral hemiarthroplasty or total hip arthroplasty due to femoral neck fracture between January 1st, 2012 and June 30th, 2019 in our center were collected. Candidate variables included demographic data, comorbidities, and routine preoperative screening tests. The main outcomes included 1-year mortality and free walking rate after hip arthroplasty. Patients were randomly divided into derivation and validation groups in the ratio of three to one. Nomograms were developed based on multivariable logistic regressions of derivation group via R language. One thousand bootstraps were used for internal validation. Those models were further tested in the validation group for external validation.

**Results:**

The final analysis was performed on 702 patients after exclusion and follow-up. All-cause 1-year mortality of the entire data set was 23.4%, while the free walking rate was 57.3%. Preoperative walking ability showed the biggest impact on predicting 1-year mortality and walking ability. Static nomograms were created from the final multivariable models, which allowed simplified graphical computations for the risks of 1-year mortality and walking ability in a certain patient. The bias-corrected C index of those nomograms for predicting 1-year mortality in the derivation group and the validation group were 0.789 and 0.768, while they were 0.807 and 0.759 for predicting postoperative walking ability. The AUC of the mortality and walking ability predicting models were 0.791 and 0.818, respectively.

**Conclusions:**

Our models enabled rapid preoperative 1-year mortality and walking ability predictions in Asian elderly femoral neck fracture patients who planned for hip arthroplasty, with adequate predictive discrimination and calibration. Those rapid assessment models could help surgeons in making more reasonable clinical decisions and subsequently reducing the risk of potential medical dispute via quantitative and individualized prognosis assessments.

## Introduction

As the worldwide population is aging, geriatric hip fracture becomes a major global public health problem. Hip fracture affects 4.5 million people per year worldwide, and the number is expected to increase to 21 million in the next 40 year s[1]. Taking up a majority of hip fractures, geriatric femoral neck fracture is a common clinical scenario encountered by orthopedic surgeons. It was widely accepted that elderly femoral neck fracture patients require hospitalization and typically warrant urgent operative treatment unless contraindicated by medical instability. Hip arthroplasty was recommended to be the first choice for both displaced and non-displaced femoral neck fractures in elderly patients (≥ 65 years). Multiple studies demonstrated that it had better clinical outcomes and long-term prognosis than internal fixation and non-surgical management [2, 3].

As early surgery (≤ 36 h after injury) for a hip fracture is recently prompted by most surgeons [4, 5], rapid preoperative assessment and clinical decision-making are requested. However, patients with hip fractures are related to high risks of functional disability and death. One third of the patients died within the first postoperative year, and hip fracture ranks among the top ten causes of disability in the elderly population [1].

In this instance, surgical decision-making could be complex and multifactorial, as poor prognosis was related to a high risk of medical dispute [6]. Surgeons have to consider not only the medically related factors but also patients’ functional expectations, economic status, and their family’s wishes [7]. Thus, rapid predicting models that allowed quantitative and individualized prognosis assessments are strongly needed as references for orthopedic surgeons during preoperative conversations. It is also beneficial for patients and their families, as they can customize care on an individual-specific level. Consequently, the purpose of the present study was to develop patient-specific factor-based nomograms, which allowed rapid preoperative predictions of 1-year mortality and walking ability in Asian elderly femoral neck fracture patients who planned for hip arthroplasty.

## Methods and material

The present study was conducted following the ethical principles of the Helsinki declaration and was approved by the institutional review board of our hospital. Signed informed consents for participation were unavailable due to the retrospective design, and the institutional review board of our hospital has waived the informed consent procedure for the present study. The electronic medical records of our hospital were reviewed to identify patients who met the following criteria: (1) those who are aged ≥ 65 years, (2) those who underwent primary unilateral hemiarthroplasty or total hip arthroplasty due to low-energy mechanism femoral neck fracture between January 1st, 2012 and June 30th, 2019. The exclusion criteria included those with (1) a previous history of trauma or surgery in the involved hip, (2) a peri-prosthetic or open fracture, (3) other injuries that required additional therapies, (4) pathological fracture, and (5) absence of intact data. We used the TRIPOD checklist when writing our report [8].

### Data collection

Data of selected patients were retrospectively retrieved from the database of our hospital. Demographic features included patients’ pre-fracture condition (residence, previous history of hip fracture in the contralateral side), time from injury to diagnosis, age, gender, marriage, medical insurance, smoking history, and cognitive status. Major comorbidities included type 2 diabetes, circulatory abnormalities (hypertension, coronary heart disease, prior myocardial infarction, and arrhythmia), chronic obstructive pulmonary disease, pulmonary infection, prior stroke, dementia, Parkinson’s disease, digestive system disorders, chronic renal failure, rheumatologic disease, and osteoporosis. Charlson comorbidity index (CCI) was calculated to obtain an overall assessment of the preoperative comorbid condition [9]. In terms of preoperative walking ability, patients were classified as free walking, need assistance, or bedridden based on the description of original medical records.

Preoperative vital signs, results of the electrocardiogram and chest radiograph, as well as blood counts and biochemical analyses (including hemoglobin, serum albumin (ALB), blood glucose, and international normalized ratio (INR)) that were obtained in the emergency department were recorded. An abnormal vital sign was defined according to the criteria of Zanker’s study [10]. Results of electrocardiogram and chest radiograph were classified as an “Abnormality” only when they were considered to be clinically significant by the correspondent authors. Treatment details including surgical procedures (hemiarthroplasty and total hip arthroplasty) and anesthesia methods (general anesthesia, peripheral nerve block, and spinal anesthesia) were collected. Perioperative major complications including aspiration pneumonia, urinary retention, deep vein thromboembolism, dislocation, periprosthetic fracture, and periprosthetic infection were also recorded from the medical record.

As for the primary outcomes, all-cause mortality and walking ability in the 1st postoperative year were obtained by telephone follow-up. Patients were classified as “free walking” when they scored 5 points and over in the locomotion section of the functional independence measure scoring system [11].

### Sample size

There is no golden standard approach to estimate the sample size requirements for risk prediction models until now. It was widely accepted to at least 10 events per candidate variable for the derivation of a risk prediction model [12]. As 21 candidate variables were included for the regression analysis, at least 210 patients in the derivation group were required for the present study.

### Statistical analysis

Continuous data were expressed as mean ± standard deviation or median with interquartile range. Categorical data was present as percent (count). The patient data set underwent a random split into derivation (75%) and validation (25%) groups, and all model creation steps were based on the derivation group only. As for the comparison between derivation and validation groups, two-sided Student’s *t* test was used for parametric variables. The difference between ratios was analyzed via Pearson’s nonparametric *χ*2 test. CCI was transformed into a binary variable, and the median of CCI (4) was set as the cutoff. Prediction models for the binary outcomes were created using multivariable logistic regression. Candidate variables included in the nomograms were identified in a screening step with the *P* values < 0.10 after multivariable logistic analysis. Odds ratios and 95% confidence intervals were calculated for each variable. The relative importance of each predictor in the model was determined by subtracting the predictor degrees of freedom from the Wald chi-square value [13].

R version 3.5.0 (R Foundation for Statistical Computing) with a specific package (rms) was utilized for all statistical testing. For the binary outcomes, each final model achieved the maximum bias-corrected concordance index (C-index). One thousand bootstrap samples were drawn to correct the bias, and the final model fit each sample. Predicted probabilities were obtained for the original sample based on each bootstrap estimated model and a C-index calculated. The bias-corrected C-index was defined as the average of these bootstrap c indices. Overall accuracy and calibration were visualized by comparing predicted versus actual probabilities, including a bias correction for overfitting. The predictive abilities of those final models were further tested in the validation group. Based on the derivation group, AUC analysis was conducted for the nomograms and Nottingham Hip Fracture Score (NHFS) in terms of 1-year mortality and walking ability. Statistical significance was set at an alpha level of 0.05. Statistical analysis was performed using the SPSS 20.0 and R software programs.

## Results

### Descriptive data

**Fig. 1:**
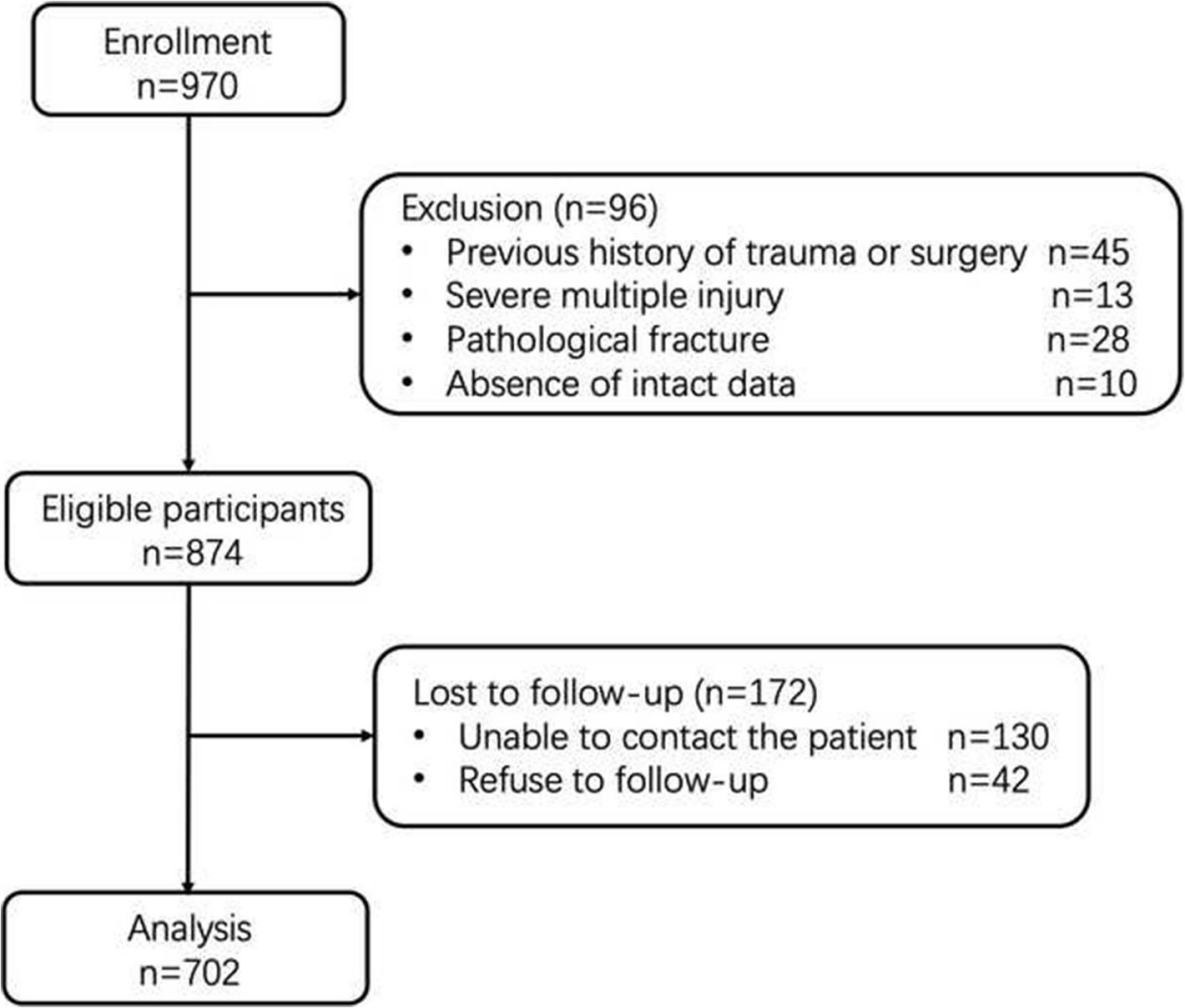
Flowchart of the present study

Nine hundred seventy patients who met the inclusion criteria were enrolled, and the final analysis was performed on 702 patients after the exclusion and follow-up. Details were shown in the flowchart (Fig. 1). The prevalence (or average) for each candidate predictor and the main outcomes in the derivation group and the validation group were calculated respectively (Table 1). No significant difference of the former-mentioned parameters was found between the derivation group and the validation group. All-cause mortality 1 year after arthroplasty of the entire data set was 23.4%, and the free walking rate was 57.3%.

**Table 1:** Descriptive statistics for main outcomes and candidate predictor variables for derivation group and validation group

Items	Derivation group (*N* = 528)	Validation group (*N* = 175)	*P*
Main outcomes			
Mortality at the 1st postoperative year (%/*n*)	22.0% (116)	27.4% (48)	0.142
Free walking rate at the 1st postoperative year (%/*n*)	58.4% (308)	53.7% (94)	0.273
Candidate predictors			
Demographic variables			
Age (Year)	77.9 ± 8.4	77.7 ± 7.6	0.774
Gender (Male)	24.5% (129)	20.0% (35)	0.225
Resident (Downtown)	82.7% (436)	82.3% (144)	0.893
Medical insurance (No)	40.2% (212)	42.3% (74)	0.631
Marriage (With spouse)	72.7% (383)	69.1% (121)	0.368
Smoking history (No)	81.2% (429)	80.6% (141)	0.843
Fragility fractures history (No)	94.5% (498)	92.6% (162)	0.352
Dementia (No)	90.7% (479)	87.4% (153)	0.211
Preoperative walking ability (ref: Bedridden)			0.847
Need assistance	25.4% (134)	27.4% (48)	
Free walking	62.9% (332)	61.7% (108)	
Surgery-related variables			
Time from injury to diagnosis (Days)	13.9 ± 20.24	17.7 ± 32.7	0.065
CCI score (≤ 4)	56.7% (299)	56.6% (99)	0.970
Vital sign (Normal)	69.6% (367)	68.0% (119)	0.684
Electrocardiogram (Normal)	38.1% (201)	38.3% (67)	0.973
Chest radiograph (Normal)	22.2% (117)	22.3% (39)	0.981
Baseline GLU (mmol/L)	6.34 ± 2.28	6.64 ± 2.64	0.153
Baseline HGB (g/L)	116.3 ± 17.7	113.9 ± 18.5	0.117
Baseline ALB (g/L)	33.7 ± 4.57	33.9 ± 4.7	0.576
Baseline INR	1.14 ± 0.97	1.10 ± 0.25	0.628
Surgical procedure (THA)	38.1% (201)	33.7% (59)	0.293
Anesthesia procedure (SA)			0.910
PNB	32.3% (170)	35.4% (62)	
GA	9.3% (49)	13.6% (21)	
Perioperative complications (No)	88.1% (465)	89.7% (157)	0.554

Note: *THA* total hip arthroplasty, *SA* spinal anesthesia, *PNB* peripheral nerve block, *GA* general anesthesia

### Predictors for all-cause mortality 1 year after arthroplasty

**Fig. 2:**
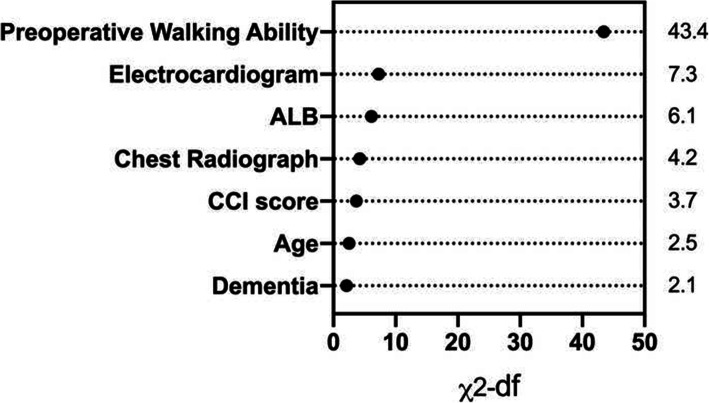
Relative importance of individual predictors within the final multivariable model for 1-year mortality was calculated from the Wald chi-square minus the predictor degrees of freedom

**Fig. 3:**
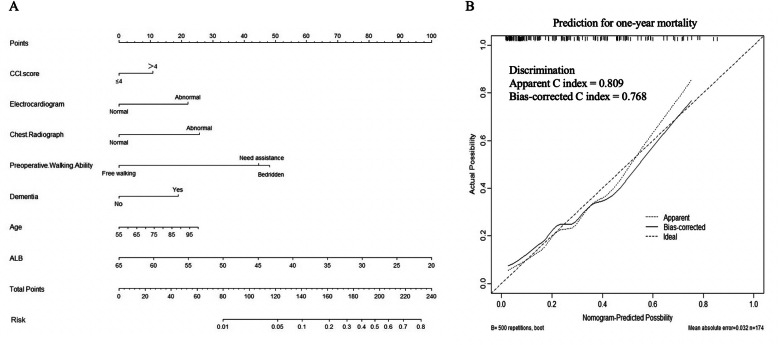
**a** Nomogram for predictive 1-year mortality. **b** Model accuracy is visualized by comparing predicted versus actual probabilities. Bias-corrected C-index for the predictive model that tested in validation group was shown.

Results of the multivariable logistic regression analysis were shown in Table 2, and odds ratios with 95% confidence intervals were calculated for each variable. According to the multivariable logistic regression analysis (significance: *P* < 0.1), 7 variables including preoperative walking ability, preoperative dementia, CCI score, age, serum ALB, electrocardiogram, and chest radiograph were selected to generate a predictive model via backward elimination. The relative predictive ability of each selected parameter was shown in Fig. 2, while preoperative walking ability led the most value. The result of the validation showed good calibration. The model accurately discriminated the risk of the patients 78.9% of the time in the derivation group (bias-corrected C-index = 0.789). The validation group (bias-corrected C-index = 0.768) showed a slightly lower C-index when tested against the final multivariable model (Fig. 3b). A static nomogram was created from the final multivariable model (Fig. 3a).

**Table 2:** Results of multivariable logistic regression analysis for mortality at the 1st postoperative year

Variables (Unit/ref)	Coefficient	Standard error	*P* value	OR	95% Confidence interval		
Age (Year)	0.036	0.019	0.06	1.037	0.998–1.077		
Gender (Male)	− 0.292	0.286	0.307	0.747	0.426–1.308		
Resident (Downtown)	0.005	0.357	0.989	1.005	0.499–2.025		
Medical insurance (No)	0.193	0.269	0.472	1.213	0.716–2.055		
Marriage (With spouse)	0.369	0.293	0.208	1.447	0.815–.569		
Smoking history (No)	1.475	0.947	0.119	4.373	0.684–27.962		
Fragility fractures history (No)	− 0.574	0.572	0.316	0.564	0.184–1.728		
Dementia (No)	0.797	0.453	0.078	0.451	0.185–1.095		
Preoperative walking ability (Bedridden)	− 1.164	0.175	0	0.312	0.222–0.44		
Time from injury to diagnosis (Days)	− 0.005	0.006	0.398	0.995	0.983–1.007		
CCI score (≤ 4)	0.596	0.275	0.03	1.815	1.059–3.111		
Vital sign (Normal)	− 0.407	0.286	0.154	0.665	0.38–1.165		
Electrocardiogram (Normal)	0.837	0.291	0.004	2.309	1.305–4.084		
Chest radiograph (Normal)	0.909	0.397	0.022	2.481	1.14–5.402		
Baseline GLU (mmol/L)	− 0.042	0.058	0.47	0.959	0.856–1.074		
Baseline HGB (g/L)	0.002	0.007	0.812	1.002	0.988–1.016		
Baseline ALB (g/L)	− 0.077	0.029	0.008	0.926	0.876–0.98		
Baseline INR	− 0.137	0.439	0.754	0.872	0.369–2.059		
Surgical procedure (THA)	0.115	0.309	0.71	1.122	0.612–2.054		
Anesthesia procedure (SA)			0.279				
PNB	0.087	0.438	0.843	1.091	0.462–2.574		
GA	0.368	0.47	0.433	0.692	0.276–1.737		
Perioperative complications (No)	− 0.168	0.372	0.652	0.845	0.408–1.752		

Note: *THA* total hip arthroplasty, *SA* spinal anesthesia, *PNB* peripheral nerve block, *GA* general anesthesia

### Predictors for walking ability 1 year after arthroplasty

**Fig. 4:**
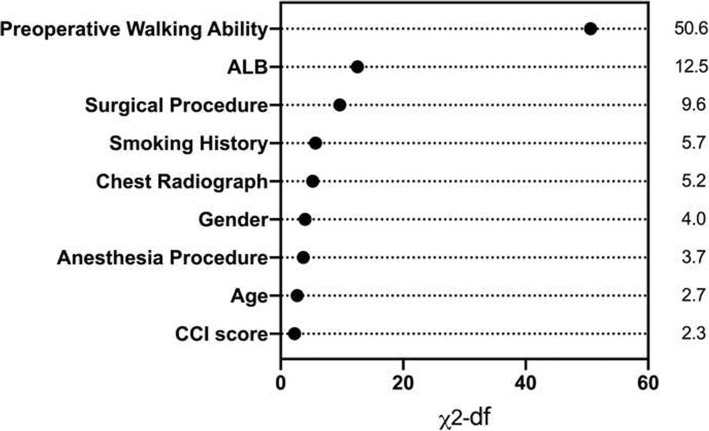
Relative importance of individual predictors within the final multivariable model for 1-year walking ability was calculated from the Wald chi-square minus the predictor degrees of freedom.

**Fig. 5:**
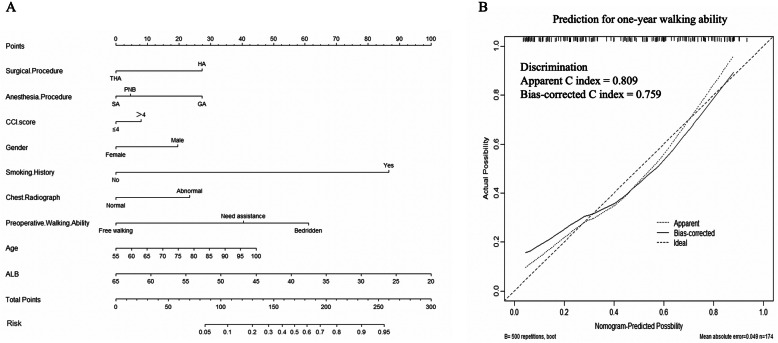
**a** Nomogram for predictive 1-year walking ability. **b** Model accuracy is visualized by comparing predicted versus actual probabilities. Bias-corrected C-index for the predictive model that tested in the validation group was shown

Results of the multivariable logistic regression analysis were shown in Table 3 and odds ratios with 95% confidence intervals were calculated for each variable. Regarding the prediction of walking ability 1 year after arthroplasty, the significant predictors (significance: *P* < 0.10) included preoperative walking ability, surgical procedure, anesthesia procedure, smoking history, gender, CCI score, age, serum ALB, and chest radiograph. As illustrated by Fig. 4, preoperative walking ability showed the biggest impact on the prediction of walking ability 1 year after arthroplasty. This model achieved adequate predictive discrimination in predicting free walking rate 1 year after arthroplasty, with bias-corrected C-index of the derivation group being 0.807. The bias-corrected C-index in the validation group was 0.759 (Fig. 5b). Nomograms were then created for the model (Fig. 5a).

**Table 3:** Results of multivariable logistic regression analysis for walking ability at the 1st postoperative year

Variables (Unit/ref)	Coefficient	Standard error	*P* value	OR	95% Confidence interval		
Age (Year)	0.032	0.017	0.054	1.032	0.999–1.066		
Gender (Male)	− 0.605	0.271	0.026	0.546	0.321–0.929		
Resident (Downtown)	− 0.52	0.322	0.106	0.594	0.316–1.117		
Medical insurance (No)	− 0.094	0.238	0.694	0.91	0.571–1.453		
Marriage (With spouse)	0.398	0.264	0.131	1.49	0.887–2.5		
Smoking history (No)	2.973	1.15	0.01	19.545	2.052–186.14		
Fragility fractures history (No)	− 0.369	0.466	0.428	0.692	0.278–1.722		
Dementia (No)	− 0.584	0.4	0.145	0.558	0.255–1.222		
Preoperative walking ability (Bedridden)	− 1.281	0.178	0	0.278	0.196–0.394		
Time from injury to diagnosis (Days)	− 0.001	0.005	0.91	0.999	0.99–1.009		
CCI score (≤ 4)	0.456	0.252	0.071	1.578	0.962–2.587		
Vital sign (Normal)	0.115	0.251	0.647	1.122	0.686–1.833		
Electrocardiogram (Normal)	0.135	0.24	0.572	1.145	0.716–1.832		
Chest radiograph (Normal)	0.732	0.293	0.013	2.08	1.17–3.695		
Baseline GLU (mmol/L)	− 0.03	0.051	0.555	0.97	0.878–1.073		
Baseline HGB (g/L)	0.009	0.006	0.142	1.009	0.997–1.022		
Baseline ALB (g/L)	− 0.094	0.025	0	0.911	0.866–0.957		
Baseline INR	− 0.114	0.226	0.612	0.892	0.573–1.389		
Surgical procedure (THA)	0.896	0.275	0.001	2.45	1.43–4.196		
Anesthesia procedure (SA)			0.059				
PNB	− 0.965	0.405	0.017	0.381	0.172–0.843		
GA	− 0.847	0.423	0.045	0.429	0.187–0.983		
Perioperative complications (No)	0.303	0.342	0.376	1.353	0.693–2.645		

Note: *THA* total hip arthroplasty, *SA* spinal anesthesia, *PNB* peripheral nerve block, *GA* general anesthesia

### Comparison between the nomograms and NHFS in terms of diagnosis efficiency

**Fig. 6:**
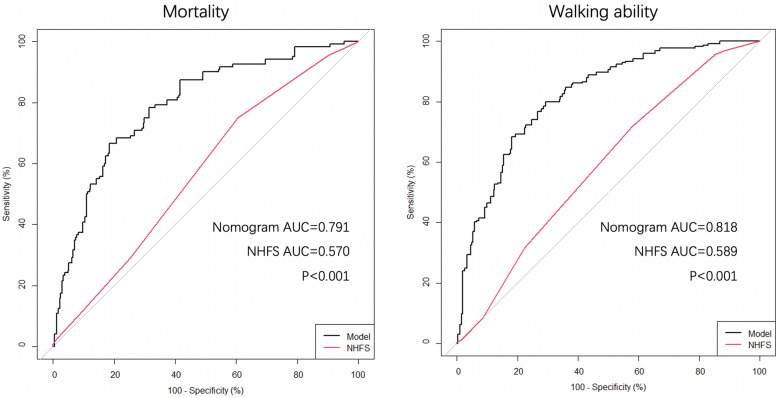
Comparison between the nomograms and NHFS in terms of AUC when predicting 1-year mortality and walking ability

As shown in Fig. 6, the nomogram for 1-year mortality prediction had a significantly higher Area under curve (AUC) when compared with the NHFS (0.791 vs 0.570, *P* < 0.001). Similar results were also found in the 1-year walking ability prediction (0.818 vs 0.589, *P* < 0.001).

## Discussion

In the present study, the all-cause mortality 1 year after arthroplasty was 23.4%. Previous studies showed that 1-year mortality of hip fracture patients varied between 16.6 to 23.9% according to different study designs [14, 15], which were consistent with ours. But we did admit that the 1-year mortality was a little higher in our study. Recently, it was reported that multidisciplinary projects provided positive effects on elderly patients who suffered from hip fracture [16]. Thus, we believed that a deep ortho-geriatric cooperation in our future clinical practice would be helpful in reducing postoperative mortality and improving long-term prognosis. Our results showed that the free walking rate 1 year after arthroplasty was 57.3%. Consistent with our study, previous studies also found that the 1-year free walking rate was approximately 40–60%, and about half of the patients did not regain their pre-fracture walking status [17, 18]. As hip fracture still ranks among the top ten causes of disability in the elderly population despite the improvement in surgical technique and multi-disciplinary care [1], further studies are strongly needed for the improvement of functional recovery.

Nomograms have been widely used in predicting clinically related outcomes after orthopedic surgery, such as a 30-day/90-day readmission [13, 19], major complications [20], periprosthetic bone loss [21], and excess cost within bundled payment [22]. To the best of our knowledge, our study represented the first time to use the nomograms in predicting mortality and walking ability of Asian elderly femoral neck fracture patients 1 year after arthroplasty. Nomogram is a pictorial representation of a complex mathematical formula designed to allow the approximate graphical computation, and points at the respective horizontal axis represented the predictive value of the variables [23]. After calculation of the total risk score based on the patients’ response for each variable, surgeons could correlate it to a specific chance of having the given outcome. The C-index in binary outcomes predicting models represents the ability to distinguish between patients who experience an event from those who do not. It is measured on a scale of 0.5 (no better than chance) to 1 (perfect discrimination). As the bias-corrected C-index for predicting mortality and walking ability in the derivation group and the validation group were both approximately 0.8, we believed that those nomograms in the present study had relatively strong discrimination according to the description of the previous study [23]. As shown in Figs. 3b and 5b, our model demonstrated slightly lower calibration in predicting 1-year mortality and free walking rate at the middle-to-low risk range. Although it was one of the limitations of our study, we believed that our models were still practical in clinical use, as predicting models are more frequently required for high-risk patients.

Several predicting models have been developed for predicting postoperative mortality and walking ability in elderly patients with femoral neck fracture until now [17, 24–27]. As the most widely used one, the NHFS showed the most promising results in predicting 30-day mortality [28]. Recently, the usage of NHFS was extended to the prediction of 1-year mortality [29] and post-discharge walking ability [27] in several studies. But unlike predicting 30-day mortality, its efficiency in 1-year mortality and walking ability prediction has not yet been fully investigated and widely accepted. Additionally, our results showed that our models for 1-year mortality (0.791 vs 0.570) and walking ability prediction (0.818 vs 0.589) had significantly higher AUC when compared with the NHFS. These poor differentiating power of NHFS in the present study is insufficient for identifying patients with high risks of poor prognosis. Other predicting models are less commonly used and are limited to neither small sample size of the derivation cohort (< 500) or lack of external validation [17, 24, 26]. The utility of some scoring systems is also limited in preoperative assessment, as their scoring items included intraoperative parameters, such as blood loss and timing of operation [25].

It is also important to note that all the former-mentioned predicting models were developed and validated according to the data retrieved from the orthopedic departments or registry centers in Europe and North America. The predictive efficiency of those models in Asian populations has not yet been evaluated. It is widely accepted that Asian countries will contribute more to the pool of hip fractures in the coming years [1]. By 2050, more than 50% of all osteoporotic fractures (including femoral neck fracture) will occur in Asia [30]. Besides, significant difference in hip fracture prevalence, bone mineral density, and bone geometry were found between the Asian and Caucasian populations [31, 32]. Thus, we proposed that our models might be more applicable for Asian elderly hip fracture patients.

Many patient-specific and surgery-related factors were reported to be closely related to increased risk of mortality and poor prognosis [33, 34]. Knowledge of these variables, however, only provides the surgeons with an individual factor that improves or worsens specific outcomes. To obtain the biggest power of discrimination and calibration, our predictive models incorporated most of the previously reported preoperative medical and socioeconomic predictors. In the present study, we found that preoperative walking ability had the biggest impact on both 1-year mortality and mobility. Similarly, other studies also claimed that preoperative walking ability was the strongest preoperative indicator of postoperative mortality in hip fractures [10, 35]. Patients who required walking assistance before fracture had a 7.5-fold higher 1-year mortality [10]. Recently, there is an increasing interest in the influence of malnutrition and dementia on long-term prognosis in patients with hip fractures. Nearly half of older patients with hip fractures are malnourished on hospital presentation [36]. Our results showed that serum ALB was predictive for both 1-year mortality and mobility, which was consistent with other studies [37, 38]. As the most commonly used biomarker of malnutrition, serum ALB level < 33 g/L was found to be a significant predictor for early mortality [39]. It was also found that nutritional supplementation effectively decreased postoperative complication rate after hip fractures [39]. Similarly, dementia was found to be an independent predictor of 1-year mortality after hip fracture [40]. Patients with preexisting dementia are more likely to experience delirium and perioperative complications during hospitalization. Interestingly, previous study and ours both reported that functional recovery was not conditioned by cognitive impairment [41]. Future studies are needed for further evaluation of the actual role of cognitive impairment on postoperative walking ability of patients.

Our study was subjected to some limitations. Firstly, although the sample size of the present study has met the requirement of the statistics, we admitted that a large-scale sample is needed for building nomograms with higher discrimination and calibration. Secondly, although the data was collected from a high-volume joint center that has a complex patient population, selection bias still existed due to the retrospective, single-center design. Thus, differences in location, medical conditions, and rehabilitation programs needed to be considered during the clinical application of the present predicting model. Besides, other variables including swallowing ability and mini nutritional assessment were found to be related to postoperative prognosis of hip fractures [42]. However, we could not enroll these variables due to the lack of original data in the medical record. Lastly, only surgically treated patients were enrolled in the present study. Researchers should be cautious when applying the present models in patients who underwent conservative treatments.

## Conclusion

Our models enabled rapid preoperative 1-year mortality and walking ability predictions in Asian elderly femoral neck fracture patients who planned for hip arthroplasty, with adequate predictive discrimination and calibration. Those rapid assessment models could help surgeons in making more reasonable clinical decisions and subsequently reducing the risk of potential medical dispute via quantitative and individualized prognosis assessments. Patients and their families could also benefit from our models, as they can customize care on an individual-specific level preoperatively. Nevertheless, differences in location, medical conditions, and rehabilitation programs needed to be considered during the clinical application of the present predicting model.

## Data Availability

The data sets used and/or analyzed during the current study are available from the corresponding author on reasonable request.

## Abbreviations

CCI: Charlson comorbidity index
ALB: Serum albumin
INR: International normalized ratio
C-index: Concordance index
NHFS: Nottingham Hip Fracture Score
AUC: Area under curve
